# Updates in Rhea: SPARQLing biochemical reaction data

**DOI:** 10.1093/nar/gky876

**Published:** 2018-10-01

**Authors:** Thierry Lombardot, Anne Morgat, Kristian B Axelsen, Lucila Aimo, Nevila Hyka-Nouspikel, Anne Niknejad, Alex Ignatchenko, Ioannis Xenarios, Elisabeth Coudert, Nicole Redaschi, Alan Bridge

**Affiliations:** 1 Swiss-Prot Group, SIB Swiss Institute of Bioinformatics, CMU, 1 rue Michel-Servet, CH-1211 Geneva 4, Switzerland; 2 Vital-IT Group, SIB Swiss Institute of Bioinformatics, Quartier Sorge, Bâtiment Génopode, CH-1015 Lausanne, Switzerland; 3 European Molecular Biology Laboratory, European Bioinformatics Institute (EMBL-EBI), Wellcome Trust Genome Campus, Hinxton, Cambridge CB10 1SD, UK; 4 Department of Biochemistry, University of Geneva, CH-1211 Geneva, Switzerland; 5 Center for Integrative Genomics, University of Lausanne, CH-1015 Lausanne, Switzerland

## Abstract

Rhea (http://www.rhea-db.org) is a comprehensive and non-redundant resource of over 11 000 expert-curated biochemical reactions that uses chemical entities from the ChEBI ontology to represent reaction participants. Originally designed as an annotation vocabulary for the UniProt Knowledgebase (UniProtKB), Rhea also provides reaction data for a range of other core knowledgebases and data repositories including ChEBI and MetaboLights. Here we describe recent developments in Rhea, focusing on a new resource description framework representation of Rhea reaction data and an SPARQL endpoint (https://sparql.rhea-db.org/sparql) that provides access to it. We demonstrate how federated queries that combine the Rhea SPARQL endpoint and other SPARQL endpoints such as that of UniProt can provide improved metabolite annotation and support integrative analyses that link the metabolome through the proteome to the transcriptome and genome. These developments will significantly boost the utility of Rhea as a means to link chemistry and biology for a more holistic understanding of biological systems and their function in health and disease.

## INTRODUCTION

Rhea (http://www.rhea-db.org) is a comprehensive and non-redundant resource of expert-curated biochemical reactions that uses chemical entities from the ChEBI ontology (1) to represent reaction participants. Rhea provides computationally tractable data on over 11 000 unique reactions curated from the scientific literature, covering reactions of the enzyme classification of the Nomenclature committee of the IUBMB (generally referred to as the Enzyme Classification, or ‘EC’) (2) as well as thousands of additional enzymatic reactions, transport reactions and spontaneously occurring reactions. Interested readers may find detailed information on Rhea reaction data in our previous publication in NAR (3).

Resources that use Rhea to describe enzymatic functions include IntEnz (4), the Enzyme Portal (5) and the Mechanism and Catalytic Site Atlas (M-CSA) (6), as well as platforms for genome scale metabolic models such as MetaNetX (7) and BiGG (8). Rhea is also currently linked to UniProtKB (9) via the enzyme classification of the IUBMB. Metabolite and metabolomics resources that use Rhea reaction data include the chemical ontology ChEBI, the SwissLipids knowledgebase for lipid biology (10) and the metabolomics repository MetaboLights (11). Rhea also links to (and is linked from) other reaction resources such as KEGG (12), MetaCyc (13) and Reactome (14), each of which also provides thousands of unique reactions.

Here, we describe recent developments in Rhea since our last publication (3), including the development of an RDF (resource description framework) representation of Rhea reaction data and a SPARQL endpoint to serve it. We also illustrate how to combine Rhea and UniProt RDF data through their respective SPARQL endpoints to generate new biological insights that combine chemical and biological knowledge from these distinct resources—a federated approach to data and knowledge mining.

## RESULTS

### Rhea RDF data model and SPARQL endpoint

In order to facilitate the integration and reuse of Rhea reaction data we have developed an RDF representation of Rhea. RDF is a core semantic web technology for the World Wide Web Consortium that is well suited to applications in distributed and decentralized environments (see https://www.w3.org/RDF/ for more details).

**Figure 1.**
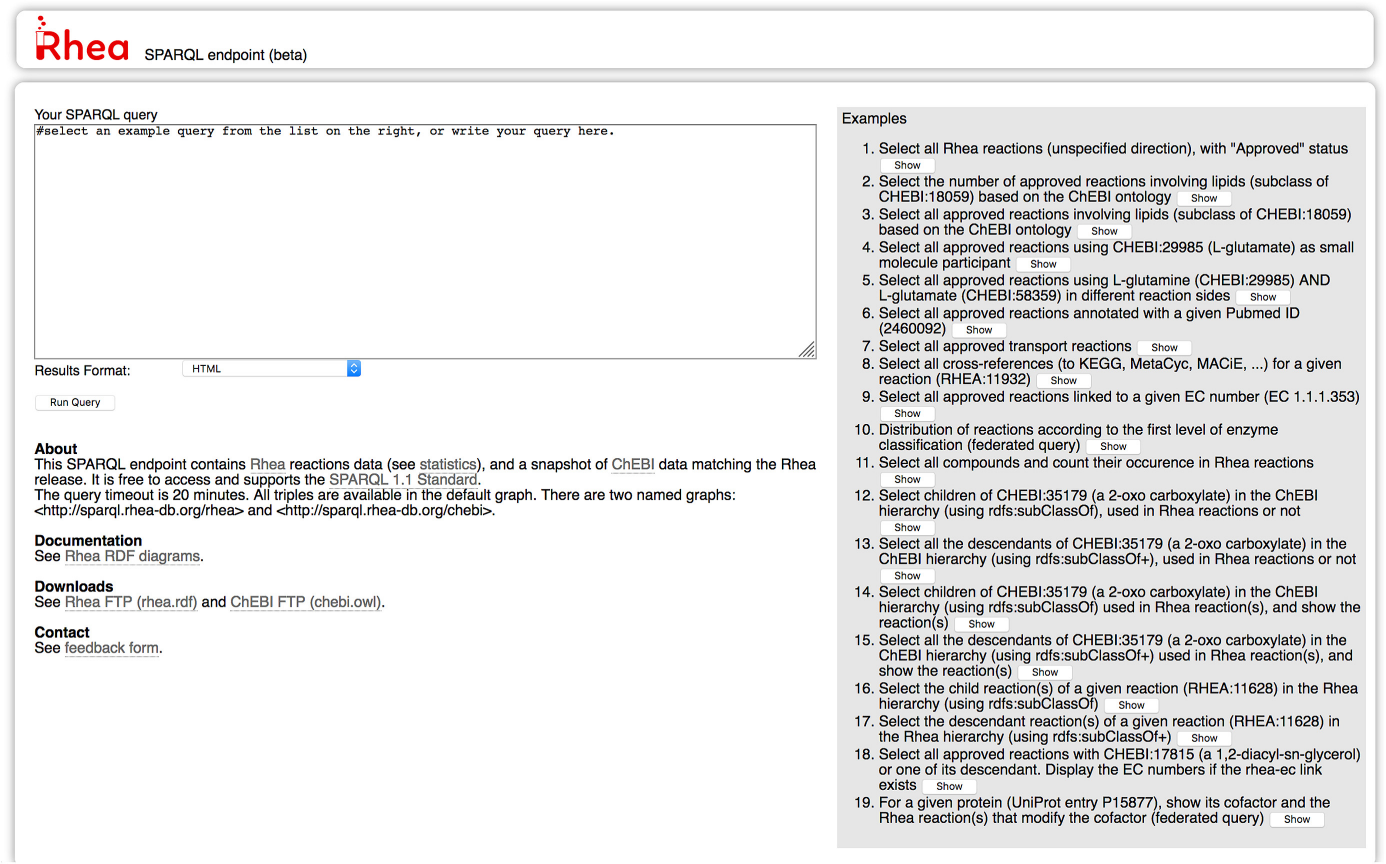
The Rhea SPARQL endpoint https://sparql.rhea-db.org/sparql. The Rhea SPARQL endpoint provides users with a portal to query Rhea RDF and other endpoints using the SPARQL 1.1 standards as well as a comprehensive set of sample queries and documentation on the Rhea RDF data model.

Users can query Rhea RDF data using SPARQL (the SPARQL Protocol and RDF Query Language) at the Rhea SPARQL endpoint https://sparql.rhea-db.org/sparql (see Figure 1), which supports a range of complex and federated queries that merge data from other SPARQL endpoints. We provide a detailed description of the Rhea data model at our website https://www.rhea-db.org/rhea_rdf_documentation.pdf and invite interested readers to consult the documentation there. The Rhea SPARQL endpoint uses Virtuoso software (https://virtuoso.openlinksw.com/) and is hosted at the Vital-IT Center for high-performance computing (https://www.vital-it.ch/) of the SIB Swiss Institute of Bioinformatics. Rhea RDF data is also available to download at ftp://ftp.ebi.ac.uk/pub/databases/rhea/rdf/ serialized as RDF/XML.

Below we provide a small number of sample federated queries that illustrate how Rhea RDF data can be combined with UniProt RDF data (at https://sparql.uniprot.org/) to generate new biological insights that are not possible using either resource alone. Each of these queries utilizes a common mapping to enzyme classes of the IUBMB to link the two resources. The Rhea SPARQL endpoint provides many more sample queries designed to help new users familiarize themselves with the Rhea RDF data model and applications.

#### Sample Rhea SPARQL Query 1. Generate a reaction network for a specified microorganism of interest

The derivation of a list of candidate metabolic functions—in the form of a network of enzymes and reactions—is one of the first steps in the construction of draft genome scale metabolic models, popular tools to simulate and study metabolic systems (15). Such draft networks would normally be the subject of further iterative improvements and curation, including compartmentalization and the addition of biomass and hypothetical reactions necessary for the model to function.

This query demonstrates the use of Rhea to construct a network of enzymes and reactions for a specific organism of interest (in this case, *Escherichia coli* strain K12), returning a list of UniProtKB proteins and the Rhea reactions they catalyze.

##### Query 1


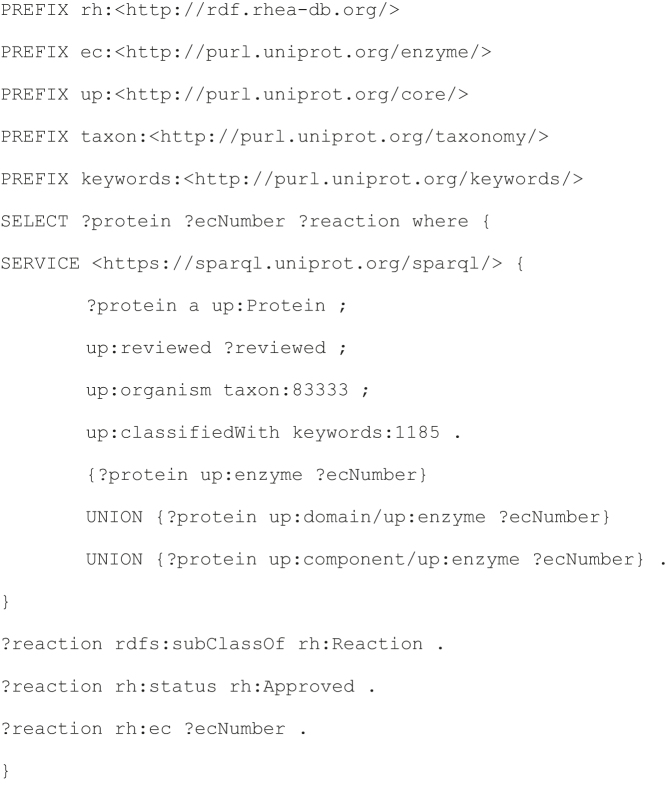


##### Query 1 result

The query returns a network of ∼1600 protein-reaction links for *E. coli*. It could be easily adapted to generate a similar draft genome scale metabolic network model for any organism with complete proteome data in UniProtKB.

#### Sample Rhea SPARQL Query 2. Link human genes, transcripts and proteins to relevant metabolites

Integrated analyses that combine metabolomics and other types of ‘omics data can advance our mechanistic understanding of disease, improve biomarker discovery and support the development personalized medicine programs (16–21).

This query demonstrates the use of Rhea to integrate knowledge of the metabolome, proteome, transcriptome and genome; it returns a list of identifiers for metabolites (ChEBI) mapped to the relevant gene and transcript (Ensembl) and protein sequences (UniProtKB/Swiss-Prot) of the enzymes that metabolize them in *Homo sapiens*. This federated query provides functionality similar to that of dedicated ID mapping tools such as MetaBridge (22).

##### Query 2


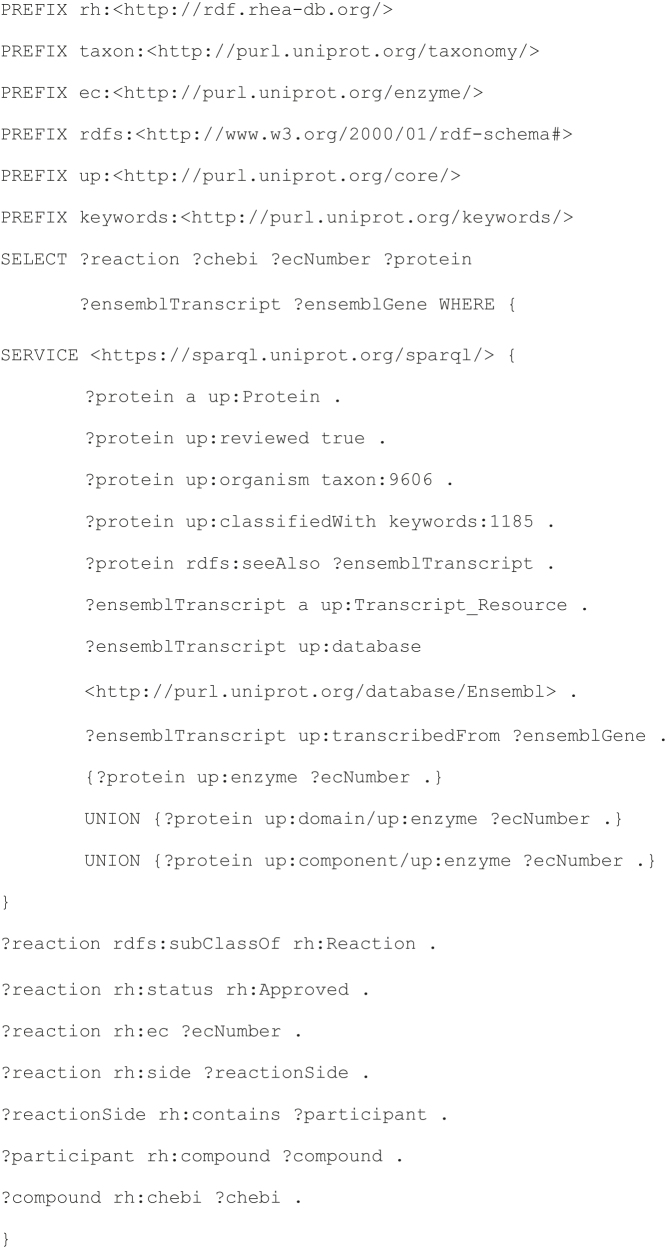


##### Query 2 result

The query currently provides ∼40 000 links between metabolites (ChEBI) through their reactions to human enzymes (UniProtKB), transcripts and genes (Ensembl). Many of the metabolites identified by this query are actually chemical classes, rather than unique chemical structures; this SPARQL query could be extended to include members of these classes too if desired, thereby generating a mapping of genes, transcripts and proteins to ‘plausible’ metabolites (according to their chemical classification by ChEBI). We provide a further example of how to leverage the ChEBI classification in the next query.

#### Sample Rhea SPARQL Query 3. Identify putative enzymes for a specific metabolite

Metabolite databases such as LIPID MAPS (23), HMDB (24) and SwissLipids (10) include a large number of metabolites for which no enzyme is currently known. Chemical classifications and classifiers (25) provide a means to improve the annotation of these uncharacterized metabolites, in much the same way that protein classifications and classifiers (typically based on homology relations) can improve the annotation of uncharacterized proteins (26).

This query demonstrates how to combine the ChEBI classification with data from Rhea and UniProtKB in order to identify candidate enzymes for a specific metabolite of interest. The metabolite in question is Δ^1^,Δ^7^-dafachronic acid (CHEBI:83137), a potent ligand for DAF-12 which regulates aging in *Caenorhabditis elegans* (27). Δ^1^,Δ^7^-dafachronic acid does not feature in any Rhea reaction and is not linked to any known enzyme. The query uses the ChEBI parent/child ontology relations to retrieve all parent ChEBI classes for Δ^1^,Δ^7^-dafachronic acid, tracing back to the root of the ChEBI ontology and then searches for the candidate enzymes and reactions for these parent classes. This query effectively extends the annotation of experimentally characterized metabolite classes in UniProtKB/Swiss-Prot to currently unannotated members of the same chemical classes.

##### Query 3


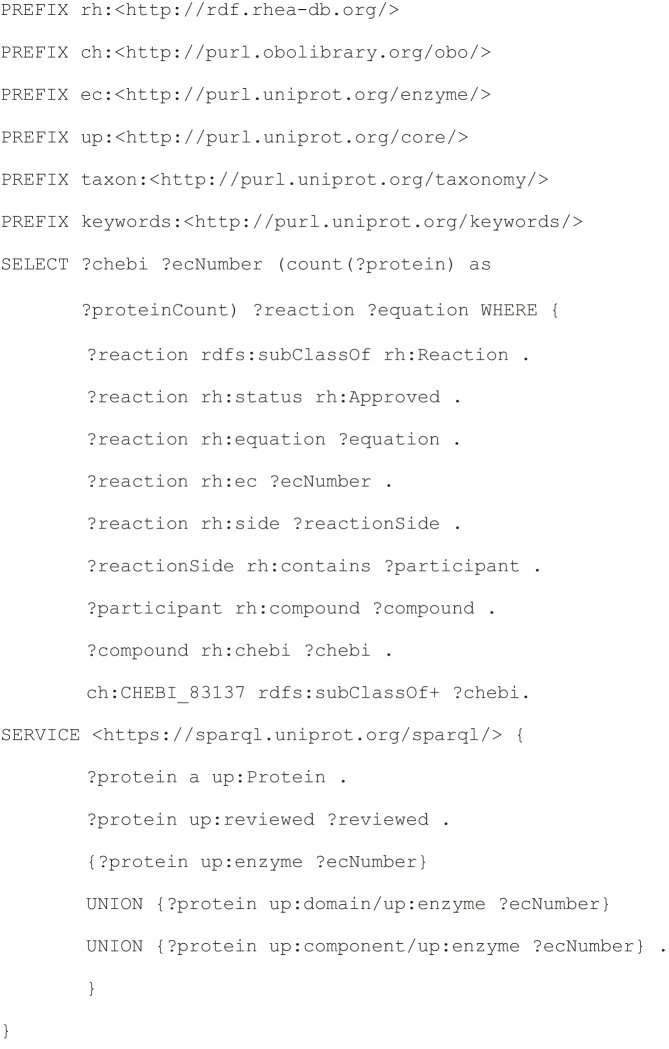


##### Query 3 result

The query proposes a total of 16 candidate enzyme classes (as defined by the enzyme classification of the IUBMB) for Δ^1^,Δ^7^-dafachronic acid. These sixteen enzyme classes act on those chemical classes of which Δ^1^,Δ^7^-dafachronic acid is a member, such as the 3-oxo-Δ^1^ steroids (CHEBI:20156) and other parent classes of increasing generality such as the 3-oxo steroids (CHEBI:47788), and its parent classes the steroids (CHEBI:35341) and ketones (CHEBI:17087). Each of these enzyme classes are potential candidates to metabolize Δ^1^,Δ^7^-dafachronic acid. Known members of the most specific of these 16 enzyme classes, EC 1.3.99.4, which catalyzes the interconversion of 3-oxo-Δ^1^ steroids and 3-oxo steroids, are currently restricted to bacteria. Members of other enzyme classes of lower specificity such as EC 1.1.1.184 (encoded by *dhrs-4* described in UniProtKB:G5EGA6), and EC 1.1.1.1 (encoded by *sodh-1, sodh-2, H24K24* and *dhs-3*, described in UniProtKB:Q17334, UniProtKB:O45687, UniProtKB:Q17335 and UniProtKB:A5JYX5) are found in *C. elegans*.

### Other modes of Rhea access

In addition to now providing the Rhea SPARQL endpoint we also continue to maintain all the modes of access (interactive searches, programmatic access and data downloads) and data formats described in our previous publication (3) at www.rhea-db.org.

### Rhea content

Rhea has continued to grow significantly since our last report through the expert curation of new chemical entities in ChEBI and reactions from peer-reviewed literature (see http://www.rhea-db.org/statistics for details). Rhea currently (release 96 of 13 July 2018) describes 11 173 unique reactions involving 9916 unique reaction participants and cites 12 611 unique literature references (PubMed identifiers). This represents an increase of ∼1900 unique reactions, 1800 unique reaction participants and 3700 literature references since our last publication (3) (which described release 75 of 30 July 2016).

## DISCUSSION

We have shown how federated SPARQL queries that combine Rhea reaction data with that from other SPARQL endpoints such as that of UniProt can facilitate a range of data integration and data mining tasks. These include the generation of draft genome-scale metabolic reaction networks and the identification of candidate enzymes, which are common use cases in systems biology applications such as metabolic modeling and engineering, and the integration of genome, transcriptome, proteome and metabolome data, which is of broad utility, including in the domain of personalized health and medicine.

The federated queries we describe currently exploit the mapping between Rhea reactions and the IUBMB enzyme classification to link Rhea and UniProtKB. In the near future UniProt will incorporate Rhea as an annotation vocabulary for enzymes in UniProtKB, and UniProt curators will directly link Rhea reactions to UniProtKB/Swiss-Prot records as part of their normal curation workflow. This will significantly increase the coverage and specificity of enzyme annotation in UniProtKB, enhancing the utility of UniProtKB and Rhea for ‘omics data integration and powering new search and analysis capabilities that combine protein sequence and function with chemical structure data.
